# Effect of Pitfall Trap Spacing on Sample Independence in Ant Community Studies in Homogeneous Grasslands (Hymenoptera: Formicidae)

**DOI:** 10.3390/biology15100762

**Published:** 2026-05-11

**Authors:** Francisco Jiménez-Carmona, Soledad Carpintero, Joaquín L. Reyes-López

**Affiliations:** Department of Botany, Ecology and Plant Physiology, University of Córdoba, Celestino Mutis Building, Rabanales Campus, 14071 Córdoba, Spain; soledadcarpintero@gmail.com (S.C.); joaquin@uco.es (J.L.R.-L.)

**Keywords:** pitfall traps, ants, trap spacing, spatial autocorrelation, pseudoreplication

## Abstract

The spacing between traps is one of the variables that can influence pitfall-trapping results. Very short distances between traps could cause pseudoreplication, which means that samples are not statistically independent. In this study, we evaluated how trap spacing affects the similarity in the composition and abundance of ant communities in two homogeneous grasslands sampled with pitfall traps separated by 0.5, 1, 2, 5, 10, 20, or 40 m. The results show that dissimilarity between the samples increases progressively with the distance between traps and that, in general, from 5 m onwards, the samples tend to be more independent of each other. These findings suggest that, in homogeneous grasslands, a distance of 5 m between pitfall traps may serve as a practical reference to reduce pseudoreplication. However, the independence threshold between samples should not be generalized directly to more heterogeneous ecosystems.

## Simple Summary

Pitfall traps are widely used to study ground-dwelling ants and other insects, but traps placed too close together may yield very similar samples. This makes it harder to know whether the results truly reflect differences in the community being studied. In this study, we tested how pitfall trap spacing affects the similarity of ant samples in two homogeneous grasslands in southern Spain. We found that traps placed very close to each other collected more similar samples, whereas differences between samples increased with distance. In general, traps separated by about 5 m produced more independent samples than traps placed at shorter distances. These results provide a practical guide for planning ant sampling in open grasslands and can help researchers improve study design and obtain more reliable results. In more complex habitats, however, the most suitable distance may be different.

## 1. Introduction

Ants (Hymenoptera: Formicidae) are eusocial insects that live in colonies [1]. The results of their sampling depend on multiple variables, including the spatial distribution of their nests, habitat type, sampling technique, and species morphology and behaviour [1,2].

The spatial distribution of nests changes with the availability of space or food resources and, as an important factor, with the presence of competing species [3]. In addition, habitat type influences both species composition and the spatial distribution patterns of their colonies [4]. Heterogeneous environments generally support more species than homogeneous ones. In the latter, there are usually fewer species, whose colonies tend to be distributed more regularly [5]. All of this will affect which species appear at a given sampling point.

Regarding sampling technique, pitfall trapping is one of the most widely used methods for sampling epigeic ants [2,6]. Each trap consists of a container—usually cup-shaped—buried flush with the soil surface and typically filled with a liquid solution that prevents the organisms from escaping. Trap features may affect which species are captured and their relative abundances, including opening diameter, depth, material, and the presence or absence of lids. The spatial arrangement of the traps is also a variable to be considered (e.g., transects, grids, or circular arrangements) [2,7]. Although a substantial body of the literature has examined some of these methodological variations and their associated biases, other variables, such as the spacing between pitfall traps, have received comparatively less attention.

When two pitfall traps are placed within a given area, they might capture similar species and abundances of individuals [8]. This could happen if the environment is homogeneous and supports a few species that are distributed in a more or less regular pattern. In such cases, spatial autocorrelation may occur, with nearby samples showing more similar values than distant ones [9,10,11]. Consequently, two closely spaced traps may capture individuals from the same nests and therefore record the same species in similar abundances. Under these conditions, samples are not independent [12] but pseudoreplicated, which may compromise the validity of the statistical inferences derived from the data [9,13]. If this occurs, statistical analyses may produce misleading results and lead to interpretations that do not reflect ecological reality [9,12,13]. One way to mitigate this issue is through careful sampling design, for example, by establishing minimum distances between sampling points that reduce spatial dependence among observations [9].

Species-specific traits, such as the size and mobility of each species or their foraging behaviour, may also influence the scale at which pseudoreplication occurs. Gotelli et al. [14] suggested that for small ant species, such as those of the genus *Temnothorax*, distances of about 2 m between pitfall traps may be sufficient to avoid pseudoreplication. However, for larger species, such as those of the genus *Camponotus*, greater distances may be required. As species composition varies across types of habitats [4], the optimal spacing between pitfall traps to avoid pseudoreplication is also likely to vary according to the type of the studied ecosystem.

Several authors have previously examined this issue. Most studies have addressed it by analyzing the number of species captured in traps as a function of trap spacing [15,16]. However, relying solely on species richness could be problematic because two sampling points may contain the same number of species while differing substantially in species identity. Consequently, multivariate approaches that consider the species composition provide a more appropriate framework for analyzing this topic, as proposed by King and Porter [17].

The aim of this study is to determine the minimum distance between pitfall traps at which the assemblages captured, considering both species composition and relative abundance, can be regarded as significantly different. To address this question, we evaluated distances between traps ranging from 0.5 to 40 m.

## 2. Materials and Methods

### 2.1. Study Area

**Figure 1:**
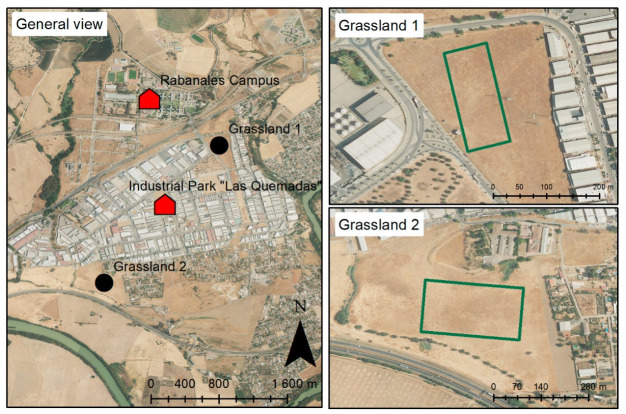
Location of the study area and sampling sites. The map shows Rabanales University Campus and the industrial area Las Quemadas (Córdoba, southern Spain). The sampling areas in both grasslands are indicated by green rectangles. These suburban grasslands were selected because of their relatively homogeneous vegetation structure and sufficient surface area to accommodate the experimental design. Extracted and adapted from Jiménez-Carmona [18].

To achieve the objectives of this study, two nearby grasslands were selected. They are located less than 2 km apart within the industrial area “Las Quemadas” in Córdoba (Spain) (Figure 1). These study sites were designated as Grassland 1 (37.909489° N, 4.714287° W), which was sampled in two periods (13–15 June 2018 and 18–20 June 2018), and Grassland 2 (37.895690° N, 4.727536° W), which was sampled in one period (12–14 June 2019).

Both sites are suburban grasslands with a relatively homogeneous appearance, dominated by nitrophilous herbaceous species and subjected to continuous grazing pressure, with a notable presence of thistle-like taxa (see Table S1). These areas were selected because they were extensive and homogeneous enough to perform the experimental design.

### 2.2. Climate

The climate of the study areas corresponds to the continental Mediterranean type (Spanish State Meteorological Agency, AEMET).

According to data from the Córdoba Airport meteorological station (AEMET), during the first sampling period for Grassland 1 (13–15 June 2018), the mean temperature was 25.1 °C (range: 14.6–37.2 °C). During the second period (18–20 June 2018), the mean temperature was 28.6 °C (range: 18.6–40.0 °C). For Grassland 2 (12–14 June 2019), the mean temperature was 22.1 °C (range: 11.8–31.3 °C). No precipitation was recorded during any sampling period.

### 2.3. Experimental Design

**Figure 2:**
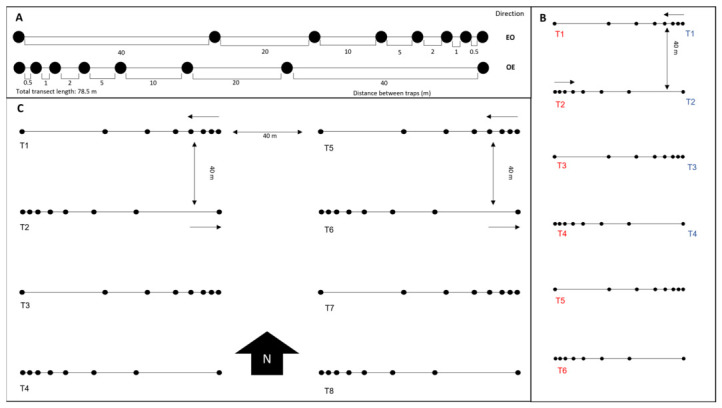
Schematic representation of the sampling design. (**A**) Layout of the pitfall trap transects, each consisting of eight traps placed at increasing distances from the starting point (0, 0.5, 1.5, 3.5, 8.5, 18.5, 38.5, and 78.5 m), resulting in distances of 0.5, 1, 2, 5, 10, 20, and 40 m between consecutive traps. (**B**) Arrangement of transects in Grassland 1. The transects placed in the first sampling period are marked in blue, while those placed in the second period are marked in red. (**C**) Arrangement of transects in Grassland 2, where traps were distributed in two blocks of four transects each, separated by 40 m. Extracted and adapted from Jiménez-Carmona [18].

In both grasslands, pitfall traps were placed along linear transects measuring 78.5 m and oriented east–west. Each transect contained eight traps. From the beginning of even-numbered transects, the traps were placed at 0 m, 0.5, 1.5, 3.5, 8.5, 18.5, 38.5, and 78.5 m. Thus, the distance between consecutive traps was 0.5 m, 1, 2, 5, 10, 20, and 40 m. The maximum distance of 40 m was selected because this scale is commonly used in studies of surface-active ant communities and has been considered sufficient to ensure sample independence without compromising spatial representativeness [19]. In odd-numbered transects, the arrangement of the traps was reversed (the first two traps were separated by 40 m) (Figure 2A). The traps were arranged this way to minimize potential effects from local environmental differences, such as variations in vegetation cover or soil characteristics.

Figure 2B shows the arrangement of the transects in Grassland 1 during the first sampling period (13–15 June 2018), with four parallel transects marked in blue, and during the second period (18–20 June 2018), with six parallel transects marked in red (80 traps in total). Figure 2C shows the arrangement of eight transects (64 traps in total) in Grassland 2, sampled from 12 to 14 June 2019 and distributed in two blocks, each with four parallel transects separated by 40 m. Trap layout was determined by the size and orientation of the sampling areas. The internal arrangement of traps within each transect was identical in both grasslands, and differences between sites concerned only the total number and spatial distribution of transects, which also depended on grazing conditions and the presence of dense patches of thorny vegetation that were not disturbed in order to avoid altering the local ant community.

Pitfall traps consisted of transparent plastic cups with a diameter of 5.7 cm at the upper opening, 5 cm at the base, a depth of 7.3 cm, and a capacity of 150 mL (Ref. 409702, Deltalab S.L., Rubí, Barcelona). Each cup was inserted into an excavated hole to ensure that the trap opening was flush with the soil surface and there were no gaps between the container and the surrounding soil.

Each trap was filled with a 1% solution of water and detergent (commercial liquid dishwashing soap) to reduce the surface tension of the water and prevent ants’ escape [7,20]. No baits or preservatives were used. This method allows recording both the presence of species and their relative abundances [21].

In all cases, traps remained active in the soil for 48 h. This enables the capture of the different species, regardless of their activity rhythm. After this period, the ants were separated from other arthropods and from detritus and preserved in ethanol. Afterwards, the ants were identified to species level by Francisco Jiménez-Carmona and Joaquín L. Reyes-López using the specialized taxonomic keys and diagnostic literature for each genus, and the number of workers per species and per trap was recorded.

### 2.4. Statistical Analyses

All statistical analyses were performed with the software R version 4.5.0 [22]. All graphs were created using the ggplot2 package [23]. Bray–Curtis dissimilarities were calculated with the vegan package [24], and linear mixed models were fitted with the lmer function from the lme4 package [25].

To evaluate the relationship between trap spacing and dissimilarity among captured ants, Bray–Curtis dissimilarities were calculated between traps within each transect using the matrix of worker abundance per species and trap. Comparisons were carried out considering the experimental distances between consecutive traps (0.5, 1, 2, 5, 10, 20, and 40 m). These distances followed an increasing sequence designed to cover both short and broad spatial scales within the same transect. Transects with substantial trap loss that prevented complete comparison across consecutive distances were excluded from the analyses.

A linear mixed model (LMM) was then fitted using Bray–Curtis dissimilarity transformed as log(Bray + 1) as the response variable to improve the approximation to normality of the residuals. The explanatory variables included Grassland 1 and Grassland 2, Trap distance—treated as a categorical variable—and the interaction between these variables. Transect was included as a random effect to account for the dependence among comparisons within the same transect. When the effect of distance was significant, post hoc multiple comparisons among distance levels were performed using estimated marginal means with Tukey adjustment.

To evaluate potential differences between short and long spatial scales, the distance variable was recoded into a new variable called Distance group, with two categories: short distances (0.5–2 m) and long distances (5–40 m). This grouping was defined after visual inspection of the graphical pattern of dissimilarity values, which suggested a marked separation between traps spaced at 0.5–2 m and those spaced at 5–40 m. A second linear mixed model with the same structure as the previous one was fitted using this variable.

Finally, although trap distance was established at fixed discrete intervals, these values represent an ordered spatial gradient. Therefore, to explore whether ant dissimilarity showed an overall distance–decay trend, a third linear mixed model was fitted, with the distance between traps included as a continuous predictor after log transformation (i.e., log(distance)) [26].

## 3. Results

A total of 3763 worker ants, belonging to 15 species, were collected in both sampling areas: fourteen species in Grassland 1 and ten in Grassland 2 (see Table 1). One transect from Grassland 2 (transect 5) had to be excluded from the analysis due to the loss of several traps. Consequently, the final number of transects analyzed was: ten in Grassland 1 (80 traps) and seven in Grassland 2 (56 traps).

The most abundant species were *Messor barbarus* (Linnaeus, 1767), *Temnothorax alfacarensis* (Tinaut & Reyes-López, 2020), *Aphaenogaster senilis* (Mayr, 1853), and *Crematogaster auberti* (Emery, 1869).

**Figure 3:**
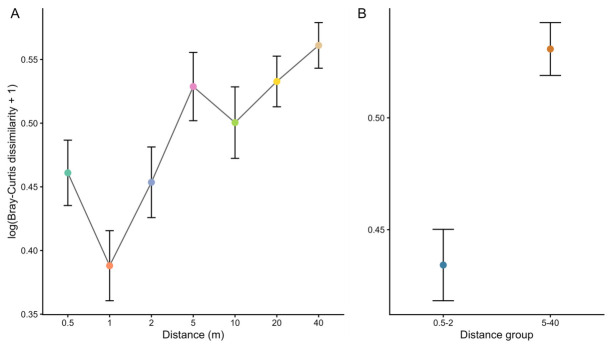
Dissimilarity between ant composition in pitfall traps separated by different distances. The values represent mean log(Bray–Curtis dissimilarity + 1) (±SE) calculated from worker abundance data. (**A**) Dissimilarity values for each distance between consecutive traps (0.5, 1, 2, 5, 10, 20, and 40 m). (**B**) Dissimilarity values grouped into two distance categories: short distances (0.5–2 m) and long distances (5–40 m). Higher values indicate greater differences in species composition and relative abundance of individuals per species between traps.

To evaluate the relationship between trap spacing and variation in ant composition and relative abundance, a dataset was constructed using Bray–Curtis dissimilarity values between consecutive traps within each transect and considering the distances defined in the experimental design (0.5, 1, 2, 5, 10, 20, and 40 m). Using these data, mean dissimilarity values between traps were calculated for each distance category and were represented graphically (Figure 3A). The graphical representation suggested the presence of two groups of trap distances with distinct patterns. On the one hand, traps separated by 0.5–2 m displayed relatively low dissimilarity values. On the other hand, traps separated by 5–40 m showed higher dissimilarity values (Figure 3B).

The first linear mixed model, which included all distance categories, indicated that trap spacing had a significant effect on Bray–Curtis dissimilarity between samples (F_6,90_ = 7.71, *p* < 0.001). In contrast, the effect of the grassland factor was not significant (F_1,15_ = 0.10, *p* = 0.75), and the interaction between grassland and distance showed a trend close to statistical significance (F_6,90_ = 2.11, *p* = 0.059; see Table S2). Post hoc multiple comparisons revealed that the shortest distances (0.5–2 m) produced significantly lower dissimilarity values than several of the larger distances, confirming the pattern observed in the graphical representation (see Table S3).

In the second model, in which the two previously defined distance groups (0.5–2 m and 5–40 m) were used, significant differences in Bray–Curtis dissimilarity values were detected between both groups (F_1,100_ = 34.53, *p* < 0.001). As in the previous model, the effect of the grassland factor was not significant (F_1,15_ = 0.014, *p* = 0.91), although a significant interaction between grassland and distance group was observed (F_1,100_ = 5.38, *p* = 0.022; see Table S2).

Finally, the model including trap distance as a continuous variable also showed a significant effect on ant dissimilarity (F_1,100_ = 29.83, *p* < 0.001), indicating that dissimilarity between traps increased progressively with the distance separating the sampling points (Table S2).

## 4. Discussion

When designing a sampling plan using pitfall traps, it is essential to understand and standardize the methodology. The distance between traps is one of the variables that may influence the results of the experiment. In fact, numerous studies attempt to avoid the potential effects of pseudoreplication caused by the proximity between samples through different statistical treatments [27,28,29]. Others address the same issue by substantially increasing the distance between traps [6,30].

Previous studies, such as those by Digweed et al. [15] and Ward et al. [16], did not reach clear conclusions regarding this issue for ants, although they conducted such for other groups such as beetles. However, these studies focused exclusively on the number of species captured, without considering which species were present or their relative abundances. By considering only species richness, the data are simplified, potentially obscuring differences that could become apparent when species composition and relative abundance are taken into account.

In this study, the results show that dissimilarity between samples increases progressively with trap spacing, revealing a distance–decay pattern in ant communities. This pattern is consistent with the general distance–decay phenomenon widely observed in ecological systems, in which nearby sampling points tend to share more species or similar abundances than more distant ones [26,31,32].

Our results also suggest the existence of two distinct distance ranges. Traps separated by 0.5–2 m show lower dissimilarity values, indicating that captures are similar at these spatial scales. In contrast, traps separated by 5–40 m show higher dissimilarity values; therefore, beyond these distances, the samples are more independent from one another.

These results are consistent with those reported by King and Porter [17], who also used pitfall traps to study ant communities in different ecosystems (forests, shrublands, and grasslands) and concluded that distances of approximately 5 m were sufficient to obtain independent samples. However, that study did not examine distances shorter than 5 m; hence, it was not possible to evaluate patterns occurring at smaller spatial scales. Our study complements those findings by including shorter distances, showing that similarity between traps can be considerable when the separation is 2 m or less. But our experimental design did not include the 2–5 m interval; consequently, we cannot assess whether samples separated by 3 or 4 m would show differences comparable to those observed at 5 m. Future studies could explore this intermediate range in greater detail to determine more precisely the threshold distance at which samples can be considered independent. Likewise, distances greater than 40 m were not included in the experimental design. Consequently, the present results do not allow us to determine whether dissimilarity stabilizes beyond that range or continues to increase at broader spatial scales. Accordingly, the 5 m threshold identified here should be interpreted as a practical lower reference for these grasslands, rather than as an upper-bound characterization of the full spatial relationship between distance and sample independence.

Besides the distance between traps, other variables may influence the presence and relative abundance of ant species in the samples, such as body size, behaviour, and foraging range [33,34,35]. Ants differ greatly in this respect. For example, ants of the genus *Cataglyphis* can travel tens or even hundreds of metres during their foraging activities [34,36]. In contrast, small species, such as those of the genus *Temnothorax*, usually forage within much smaller areas around the nest, typically within the immediate surroundings of the colony [1,35,37]. Such differences could influence the spatial scale at which samples obtained with pitfall traps can be considered independent.

Another important variable to consider is the complexity of the habitat. The present study examined only one of the ecosystem types analyzed by King and Porter [17], the grassland. It is therefore necessary to determine whether the minimum distance required to avoid pseudoreplication could differ in more complex ecosystems. In addition, the spatial extent of the sampled area may also influence the practical suitability of a given trap spacing [31,32]. Numerous studies have shown that increased vegetation structural complexity is often associated with higher ant diversity [38,39,40,41,42,43]. Moreover, habitat structure itself may affect pitfall-trap trappability, potentially introducing bias when samples are compared across structurally contrasting microhabitats [44]. In more heterogeneous environments, the presence of different microhabitats—such as areas with unequal vegetation cover, presence or absence of litter, or proximity to trees—may promote greater species turnover at smaller spatial scales [31]. Therefore, the minimum distance between traps required to obtain independent samples could be smaller than that estimated in our study. In any case, if experimental requirements or site characteristics require shorter trap spacing, different statistical approaches can be used to control for dependence among samples, such as linear mixed models or other methods that account for the hierarchical or spatial structure of the data [13,27,45,46].

For this reason, the present results should be interpreted as a practical reference specifically for relatively homogeneous grasslands, where vegetation structure and environmental conditions vary comparatively little at short spatial scales. In more heterogeneous habitats, greater small-scale variation in microhabitat conditions may alter the rate at which similarity declines with distance [31,32,38,39,40,41,42,43]. Hence, the threshold observed here should not be extrapolated directly without preliminary evaluation.

In summary, our results indicate that, in relatively homogeneous grasslands, a distance of approximately 5 m between pitfall traps may serve as a practical reference for obtaining more independent samples. However, this threshold could be refined by future studies, including distances between 2 and 5 m, which were not evaluated in the present experimental design. In any case, this distance can be influenced by the structural complexity of the habitat and by the species composition of the community. Consequently, in studies conducted in other ecosystems, it would be advisable to conduct preliminary evaluations to determine the most appropriate spacing between pitfall traps.

## 5. Conclusions

In relatively homogeneous grasslands, increasing the distance between pitfall traps reduces the similarity between samples and improves sample independence. In this study, a spacing of about 5 m provided a practical lower reference for obtaining more independent ant samples. However, this threshold may vary in more heterogeneous habitats and should be assessed in other ecosystems.

## Figures and Tables

**Table 1:** Ant species recorded in the study and the number of workers captured in pitfall traps in each sampling area (Grassland 1 and 2).

Species		Grassland 1 (*n* Workers)	Grassland 2 (*n* Workers)	Total Workers
*Messor barbarus*	(Linnaeus, 1767)	301	661	962
*Temnothorax alfacarensis*	(Tinaut and Reyes-López, 2020)	528	0	528
*Aphaenogaster senilis*	(Mayr, 1853)	220	197	417
*Crematogaster auberti*	(Emery, 1869)	19	353	372
*Tetramorium forte*	(Forel, 1904)	294	0	294
*Tetramorium semilaeve*	(André, 1883)	34	212	246
*Cataglyphis velox*	(Santschi, 1929)	132	108	240
*Tapinoma nigerrimum* complex		3	194	197
*Cataglyphis rosenhaueri*	(Santschi, 1925)	66	126	192
*Goniomma hispanicum*	(André, 1883)	32	82	114
*Plagiolepis pygmaea*	(Latreille, 1798)	103	0	103
*Camponotus micans*	(Nylander, 1856)	46	37	83
*Cardiocondyla batesii*	(Forel, 1894)	0	7	7
*Temnothorax tyndalei*	(Forel, 1909)	6	0	6
*Temnothorax racovitzai*	(Bondroit, 1918)	2	0	2
Total workers (N)		1786	1977	3763
Number of species (S)		14	10	15

## Data Availability

The data supporting the results of this study are available from the corresponding author upon reasonable request. They are not publicly available at this stage.

## Supplementary Materials

The following supporting information can be downloaded at https://www.mdpi.com/article/10.3390/biology15100762/s1. Table S1: List of plant species recorded in the study grasslands; Table S2: Summary of linear mixed models testing the effect of trap spacing on ant dissimilarity; Table S3: Significant Tukey post hoc comparisons among trap distances within each grassland (Model 1).
