# Supplementary material for: Effect of Pitfall Trap Spacing on Sample Independence in Ant Community Studies in Homogeneous Grasslands (Hymenoptera: Formicidae)
Source: Biology (Basel). 2026 May 11;15(10):762. doi: 10.3390/biology15100762 (PMC13203251; doi:10.3390/biology15100762)
Supplement: Supplementary file 1 [file biology-15-00762-s001.zip › biology-4261552-supplementary.pdf]

Table S1. List of plant species recorded in the study grasslands.

Species	Presence in Grassland 1	Presence in Grassland 2
Aegilops geniculata Roth	X	
Andryala integrifolia L.	X	X
Astragalus pelecinus (L.) Barneby		X
Atractylis cancellata L.	X	
Avena sterilis L.	X	X
Bellardia trixago (L.)All	X	
Bromus hordeaceus L.	X	
Bromus spp.	X	X
Cardus bourgeanus Boiss. & Reuter		X
Carlina corymbosa L.	X	X
Chondrilla juncea L.	X	X
Cichorium intybus L.	X	X
Convolvulus arvensis L.	X	X
Cynara humilis L.	X	
Cynodon dactylon (L.) Pers.	X	X
Cynoglossum creticum Mill.	X	X
Daucus carota L.	X	X
Echium plantagineum L.	X	X
Eryngium campestre L.	X	
Foeniculum vulgare Mill.	X	X
Galactites tomentosa Moench	X	X
Hordeum sp.		X
Lactuca serriola L.		X
Marrubium vulgare L.		X
Medicago orbicularis (L.) Bartal.	X	
Medicago polymorpha L.	X	X
Ononis spp.	X	X
Onopordum nervosum Boiss.		X
Pallenis spinosa (L.) Cass.	X	
Paspalum paspalodes (Michx) Scribner	X	X
Phalaris canariensis L.	X	X
Plantago lagopus L.	X	X
Pulicaria paludosa Link		X
Rumex pulcher L.	X	X
Salvia verbenaca L.		X
Scolymus hispanicus L.		X
Silybum marianum (L.) Gaertner		X
Sinapis sp.		X
Torilis spp.	X	X
Trifolium angustifolium L.	X	X
Trifolium campestre Schreb. in Sturm	X	X
Trifolium stellatum L	X	
Verbascum sinuatum L.	X	X
Verbena officinalis L.		X
**Total species**	**20**	**23**

Table S2. Summary of linear mixed models testing the effect of trap spacing on ant dissimilarity

Model	Term	NumDF	DenDF	F	p
Model 1: Grassland x trap distance	Grassland	1	15	0.10	0.7505
Model 1: Grassland x trap distance	Trap distance	6	90	7.71	<0.001
Model 1: Grassland x trap distance	Grassland x trap distance	6	90	2.11	0.0596
Model 2: Grassland x distance group	Grassland	1	15.233	0.01	0.9067
Model 2: Grassland x distance group	Distance group	1	100	34.53	<0.001
Model 2: Grassland x distance group	Grassland x distance group	1	100	5.38	0.0224
Model 3: Grassland x log(trap distance)	Grassland	1	28.909	0.43	0.5187
Model 3: Grassland x log(trap distance)	log(trap distance)	1	100	29.83	<0.001
Model 3: Grassland x log(trap distance)	Grassland x log(trap distance)	1	100	2.98	0.0875

Table S3. Significant Tukey post hoc comparisons among trap distances within each grassland (Model 1)

Grassland	Comparison	Estimate	SE	df	t ratio	adjusted p
Grassland 1	1 m vs 40 m	-0.1611	0.0405	90	-3.979	0.0026
Grassland 1	10 m vs 40 m	-0.1239	0.0405	90	-3.061	0.0443
Grassland 2	1 m vs 5 m	-0.1883	0.0484	90	-3.891	0.0035
Grassland 2	1 m vs 10 m	-0.2198	0.0484	90	-4.542	0.0003
Grassland 2	1 m vs 20 m	-0.2115	0.0484	90	-4.370	0.0006
Grassland 2	1 m vs 40 m	-0.1900	0.0484	90	-3.925	0.0031
